# IL-6 Mutation Attenuates Liver Injury Caused by *Aeromonas hydrophila* Infection by Reducing Oxidative Stress in Zebrafish

**DOI:** 10.3390/ijms242417215

**Published:** 2023-12-07

**Authors:** Wenya Zhai, Zhensheng Wang, Canxun Ye, Lan Ke, Huanling Wang, Hong Liu

**Affiliations:** 1 Key Lab of Freshwater Animal Breeding, College of Fisheries, Ministry of Agriculture and Rural Affair/Key Laboratory of Agricultural Animal Genetics, Breeding and Reproduction, Ministry of Education, Huazhong Agricultural University, Wuhan 430070, China; zhaiwenya1999@163.com (W.Z.); wangzhensheng@webmail.hzau.edu.cn (Z.W.); 2018308210107@webmail.hzau.edu.cn (C.Y.); 15623196532@163.com (L.K.); hbauwhl@hotmail.com (H.W.); 2 Engineering Research Center of Green Development for Conventional Aquatic Biological Industry in the Yangtze River Economic Belt, Ministry of Education, Wuhan 430070, China

**Keywords:** CRISPR/Cas9, interleukin 6, bacterial infection, transcriptome, reactive oxygen species

## Abstract

Interleukin-6 (IL-6), a pleiotropic cytokine, plays a crucial role in acute stress induced by bacterial infection and is strongly associated with reactive oxygen species (ROS) production. However, the role of IL-6 in the liver of fish after *Aeromonas hydrophila* infection remains unclear. Therefore, this study constructed a zebrafish (*Danio rerio*) *il-6* knockout line by CRISPR/Cas9 to investigate the function of IL-6 in the liver post bacterial infection. After infection with *A. hydrophila*, pathological observation showed that *il-6^−/−^* zebrafish exhibited milder liver damage than wild-type (WT) zebrafish. Moreover, liver transcriptome sequencing revealed that 2432 genes were significantly up-regulated and 1706 genes were significantly down-regulated in *il-6^−/−^* fish compared with WT fish after *A. hydrophila* infection. Further, gene ontology (GO) analysis showed that differentially expressed genes (DEGs) were significantly enriched in redox-related terms, including oxidoreductase activity, copper ion transport, etc. Kyoto Encyclopedia of Genes and Genomes (KEGG) analysis showed that DEGs were significantly enriched in pathways such as the PPAR signaling pathway, suggesting that *il-6* mutation has a significant effect on redox processes in the liver after *A. hydrophila* infection. Additionally, *il-6^−/−^* zebrafish exhibited lower malondialdehyde (MDA) levels and higher superoxide dismutase (SOD) activities in the liver compared with WT zebrafish following *A. hydrophila* infection, indicating that IL-6 deficiency mitigates oxidative stress induced by *A. hydrophila* infection in the liver. These findings provide a basis for further studies on the role of IL-6 in regulating oxidative stress in response to bacterial infections.

## 1. Introduction

Cytokines are a class of small molecule proteins with a wide range of biological activities [1], such as activating the immune response to resist the invasion of pathogenic microorganisms. Among them, interleukin 6 (IL-6), a typical interleukin factor, plays pleiotropic roles in immune response, lipid metabolism, and haematopoiesis [2].

Inflammation, a crucial component of the body’s defense mechanism, serves to protect the host against exogenous pathogens [3]. However, previous studies have shown that inflammation induced by immune cytokines can cause tissue damage [4]. Flavonoids provide neuroprotection by inhibiting the production of inflammatory mediators including TNF, IL-1, and IL-6 [5]. Under normal physiological conditions, the levels of IL-6 in tissues and plasma are typically low. However, IL-6 expression increases rapidly and substantially when stimulated by pathogenic microorganisms such as bacteria, fungi, and viruses [6]. Numerous studies have shown that overproduction of IL-6 triggers a cytokine storm that leads to excessive inflammation in the body [7]. Moreover, tissue damage due to cytokine storms was significantly attenuated when IL-6 signaling was inhibited [8]. Therefore, IL-6 is regarded as an important inducer of cytokine storms.

IL-6 plays a pivotal role in the acute phase response of the liver. However, uncontrolled immunity, triggered by acute phase factors, can lead to liver injury [9]. IL-6 induces the expression of IL-17 and promotes hepatocyte apoptosis, thus aggravating liver damage [10]. Li’s study suggests that reducing IL-6 is beneficial to attenuating ethanol-induced liver injury [11]. In septic mice, *IL-6* knockdown attenuated skeletal muscle atrophy by inhibiting mitochondrial reactive oxygen species (ROS) production in skeletal muscle [12]. This implies that IL-6 may be closely related to ROS production. Mitochondria are considered a significant source of ROS [13]. The liver is a major target for ROS due to the fact that hepatocytes contain relatively more mitochondria compared with other cell types [14]. ROS, as products of oxidative processes, play an essential role in the host’s defense against bacterial infection [15]. However, excessive ROS has detrimental effects on cellular components such as proteins, lipids, and DNA, causing an oxidation-reduction (redox) imbalance and inducing oxidative stress, which consequently leads to organ and tissue damage [16]. Increased ROS leads to hepatic ischemia/reperfusion injury in the endotoxemia model [17]. The use of ROS scavengers can inhibit hepatic oxidative stress, thereby reducing liver injury induced by exposure to trichloroethylene [18]. Mitochondria-targeted antioxidants significantly reduced cell death and mitochondrial injury in ischemia-reperfusion rats [19]. These studies suggest a strong link between ROS and tissue damage. Therefore, further exploration of the association between IL-6 and ROS is warranted.

*Aeromonas hydrophila*, a Gram-negative bacterium commonly found in aquatic environments [20], causes motile aeromonad septicemia in a variety of farmed fish species, resulting in significant economic losses for the global aquaculture industry. *A. hydrophila* infection can lead to inflammation, apoptosis, and oxidative stress, and ultimately reduce the resistance and survival rate of fish. Previous studies have demonstrated that reducing oxidative stress can increase the resistance to *A. hydrophila* infection in diverse fish species, including zebrafish (*Danio rerio*) [21]. Therefore, further exploration into the regulatory pathways of oxidative stress may hold the potential to improve the ability of fish to resist bacterial infection.

IL-6 can be strongly induced by *A. hydrophila* in various species of fish [21,22]. It has been shown that IL-6 activates the JAK2/STAT3 signaling pathway in fish [23]. JAK2/STAT3 plays an important role in the regulation of various cellular life processes such as apoptosis, growth, cycling, and inflammatory response [24]. However, no previous study has investigated the relationship between IL-6 and liver injury induced by bacterial infection in fish. Therefore, in this study, zebrafish was selected as the model organism, and CRISPR/Cas9 technology was used to construct an *il-6* knockout zebrafish line. Since IL-6 is closely related to ROS production, we hypothesized that IL-6 deficiency may affect the level of redox after *A. hydrophila* infection, thereby altering the degree of liver injury. This finding aspires to expedite the elucidation of antimicrobial mechanisms in fish, thereby advancing our understanding of host responses to microbial challenges.

## 2. Results

### 2.1. Generation of il-6^−/−^ Zebrafish

**Figure 1:**
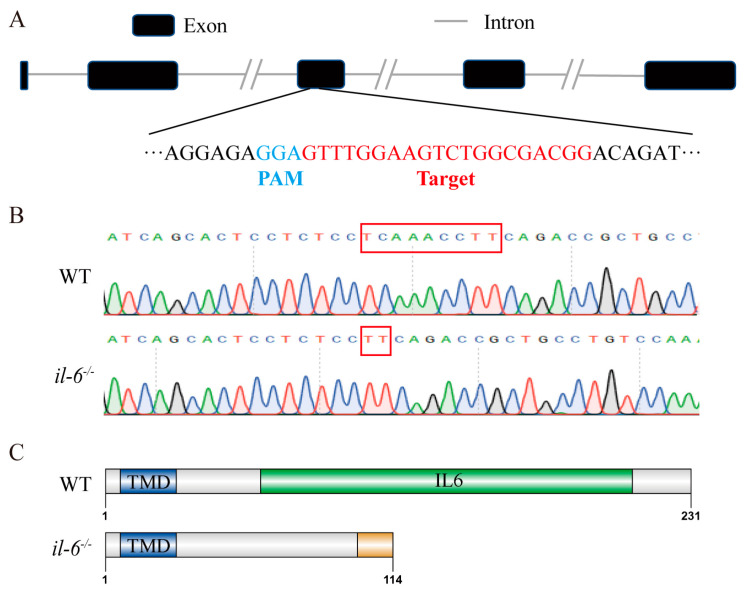
Generation of *il-6^−/−^* zebrafish via CRISPR/Cas9 technology. (**A**) Diagram of the IL-6 single-guide RNA (sgRNA). Boxes depict exons while lines depict introns. Protospacer adjacent motif (PAM) and target sequences are indicated in blue and red, respectively. (**B**) Sequencing results of wild-type (WT) and *il-6* knockout homozygous (*il-6^−/−−/−^*) zebrafish. The red frame indicates base deletion. According to the DNA sequencing results, *il*-6^−/−^ zebrafish with 7 bp deletion mutation were obtained. (**C**) Protein domains of the *il-6* gene in WT and *il-6^−/−^* zebrafish. The TMD domain is shown in blue, the IL6 domain in green, and the mutated region of the protein in yellow. *il*-6^−/−^ zebrafish showed a frameshift mutation in IL-6, resulting in premature termination of translation compared to wild-type zebrafish.

To investigate the function of IL-6 following *A. hydrophila* infection, *il*-*6*^−/−^ zebrafish was generated by CRISPR/Cas9 technology. A single-guide RNA (sgRNA) that selectively targets exon 3 of the *il*-6 gene (Figure 1A) was designed and injected with purified Cas9 protein into the one-cell stage zebrafish embryos. Through crossing F0-positive zebrafish with WT zebrafish, various mutations in the *il*-6 gene were discerned. Finally, a mutation with 7 bp deletion was identified and selected to establish *il*-6^−/−^ zebrafish. The genotypes of both WT and *il-6^−/−^* zebrafish were subsequently confirmed by sequencing (Figure 1B). The 7 bp deletion led to a frameshift mutation, causing premature termination of translation and concomitant absence of the IL6 domain (Figure 1C). In addition, we meticulously recorded the count of adult fish belonging to the categories of wild type, heterozygous, and homozygous in F2. The observed distribution of genotypic frequencies closely followed Mendelian inheritance laws (Figure S1), indicating the absence of any survival adversity in *il-6^−/−^* zebrafish.

### 2.2. Effect of il-6 Mutation on Liver Injury Caused by A. hydrophila

**Figure 2:**
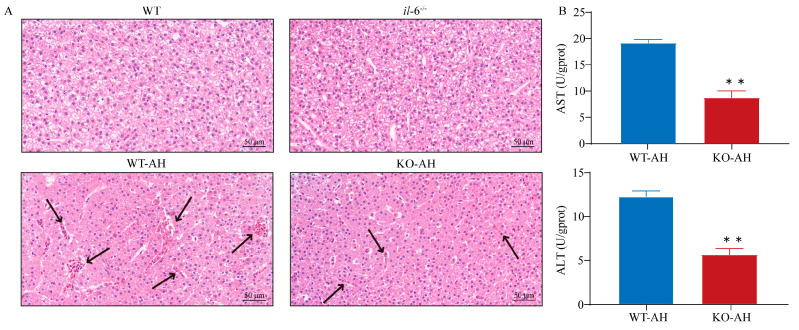
Liver injury in WT and *il-6^−/−^* zebrafish post *A. hydrophila* infection. (**A**) Pathological changes in WT and *il-6^−/−^* zebrafish liver tissue after *A. hydrophila* infection were indicated by Hematoxylin and Eosin (HE) staining. Samples were collected at 12 h after *A. hydrophila* infection. The black arrow indicates infiltration of red blood cells. Scale bar = 50 μm. *il-6^−/−^* zebrafish had milder liver injury compared to WT zebrafish after *A. hydrophila* infection. (**B**) Liver alanine transaminase (ALT) and aspartate transaminase (AST) levels. Samples used for enzyme activity (biological replicates = 5, experimental repetitions = 3) were collected 12 h after *A. hydrophila* infection. Data were subjected to the Student’s *t*-test to identify the significance. The levels of AST and ALT in the liver of *il-6^−/−^* zebrafish were significantly lower than those of WT zebrafish. ** *p* < 0.01. WT-AH: WT zebrafish treated with *A. hydrophila*; KO-AH: *il-6^−/−^* zebrafish treated with *A. hydrophila*.

In order to evaluate the impact of *il-6* mutation on liver injury caused by *A. hydrophila* stress, both WT and *il*-*6^−/−^* zebrafish were subjected to intraperitoneal injection of 1 µL *A. hydrophila* (2.5 × 10^5^ CFU/mL) and symptoms were monitored. Histopathological examination of liver tissues from WT and *il-6^−/−^* zebrafish before and after *A. hydrophila* infection was performed by HE staining to assess and identify liver injury (Figure 2A). The observation of histological sections revealed that both WT and *il-6^−/−^* zebrafish displayed significant liver injury at 12 h post *A. hydrophila* infection. In addition, pathologic abnormalities and morphological damage in livers were extensively attenuated in *il*-*6*^−/−^ zebrafish compared with WT zebrafish. Furthermore, the levels of aspartate aminotransferase (AST) and alanine aminotransferase (ALT) in the liver of *il*-*6*^−/−^ zebrafish were significantly lower than those of WT zebrafish (** *p* < 0.01; Figure 2B).

### 2.3. Identification of DEGs in the Liver Transcriptome of WT and il-6^−/−^ Zebrafish after A. hydrophila Infection

**Figure 3:**
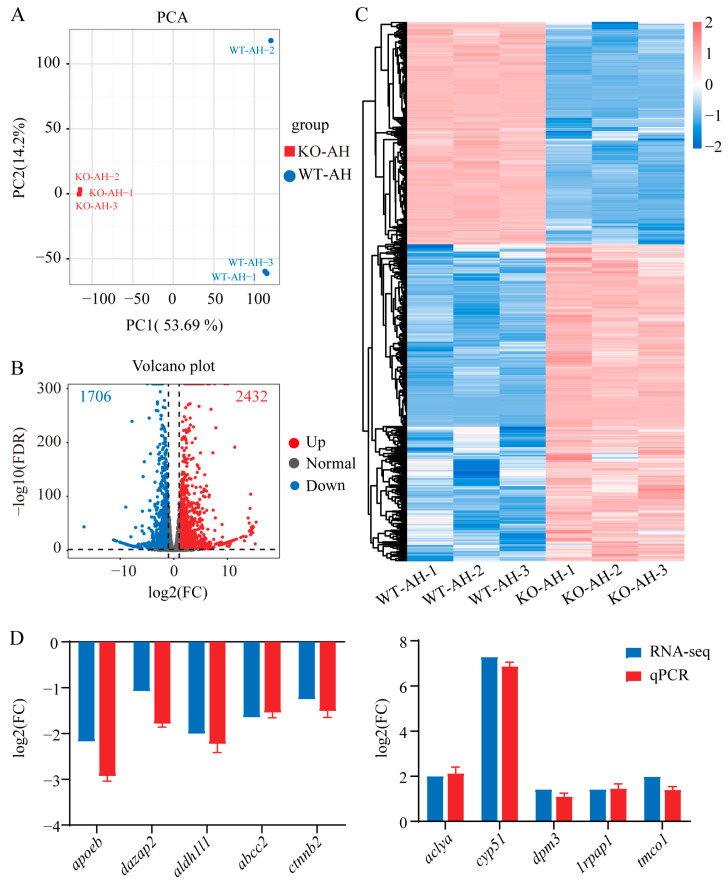
Analysis and qPCR validation of liver transcriptome data from the WT and *il-6^−/−^* zebrafish after *A. hydrophila* infection. (**A**) Principal component analysis (PCA) on the liver transcriptome data of WT and *il-6^−/−^* zebrafish after *A. hydrophila* infection. Volcano plots (**B**) and heatmap clustering (**C**) of differentially expressed genes (DEGs). The points of the blue represent down-regulated genes, the points of the red represent up-regulated genes, and the gray points represent genes without significant change. The results showed that the transcriptome of the KO-AH group was significantly different from that of the WT-AH group. (**D**) Validation of transcriptome data using qPCR. Ten genes were randomly selected. The relative expression levels were identified by qPCR (blue column) and plotted against the expression levels from the transcriptome data (red column). The qPCR results demonstrated strong concordance with the transcriptome data, confirming the reliability of the transcriptome data. *apoeb*: apolipoprotein Eb; *dazap2*: azoospermia-associated protein 2; *aldh1l1*: aldehyde dehydrogenase 1L1; *abcc2*: ATP-binding cassette-2; *ctnmb2*: catenin, beta 2; *aclya*: ATP citrate lyase a; *cyp51*: sterol 14α-demethylase; *dpm3*: dolichol-phosphate-mannose-3; *lrpap1*: low-density lipoprotein receptor-related protein-associated protein 1; *tmco1*: transmembrane and coiled-coil domains 1. WT-AH: WT zebrafish treated with *A. hydrophila*; KO-AH: *il-6^−/−^* zebrafish treated with *A. hydrophila*.

RNA-seq was performed on liver samples from both WT and *il*-*6*^−/−^ zebrafish after *A. hydrophila* infection. An average of 24,501,118 reads (WT-AH) and 24,649,957 reads (KO-AH) were obtained, and detailed statistics were listed in Table S1. The percentage of clean reads successfully mapped to the zebrafish reference genome ranged from 92.23% to 94.11%, with an average alignment rate of 93.19%. The principal component analysis (PCA) showed that the transcriptome of the KO-AH group was significantly different from that of the WT-AH group (Figure 3A).

A comparison between KO-AH and WT-AH groups resulted in the identification of 4138 differently expressed genes (DEGs, adjusted FDR < 0.01 and fold change ≥ 2). The volcano plot revealed that 1706 genes were significantly down-regulated and 2432 genes were significantly up-regulated (Figure 3B). The global gene expression profiles showed a substantial distinction between WT-AH and KO-AH groups (Figure 3C). Subsequently, 10 genes were randomly selected for validation of the transcriptomic data through qPCR (Figure 3D). The qPCR results demonstrated strong concordance with the transcriptome data, confirming the reliability of the transcriptome data.

### 2.4. Effect of il-6 Mutation on Oxidative Stress Caused by A. hydrophila in the Liver

**Figure 4:**
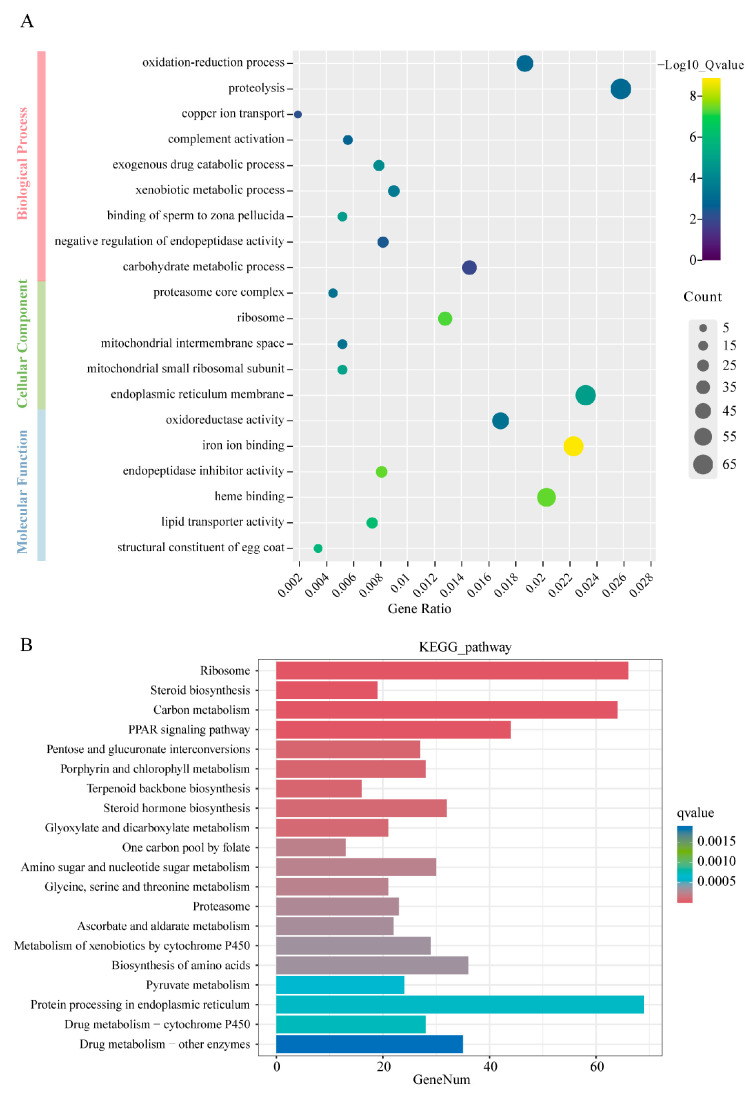
GO and KEGG pathway enrichment analysis of DEGs. (**A**) GO annotation analysis. Red: biological process; green: cellular component; blue: molecular function. The vertical axis represents the name of the GO term, the horizontal axis represents the gene ratio, the size of the dots indicates the number of differentially expressed genes in the term, and the color of the dots corresponds to different q-value ranges. (**B**) Top 20 pathways enriched in KEGG of DEGs. The vertical axis represents the name of the pathway, the horizontal axis represents the number of differentially expressed genes in the pathway, and the color corresponds to different q-value ranges.

In order to further investigate the impact of *il-6* mutation on gene expression in zebrafish liver following *A. hydrophila* infection, Gene Ontology (GO) enrichment analysis was performed on DEGs (Figure 4A). A range of notable biological processes were observed, mainly including the redox process (GO:0055114), proteolysis (GO:0006508), and copper ion transport (GO:0006825). Remarkably, one of the most representative molecular functions of these DEGs was oxidoreductase activity (GO:0016491). To further investigate the functional implications of these findings, we performed a Kyoto Encyclopedia of Genes and Genomes (KEGG) pathway analysis, which identified 43 pathways that showed statistical significance (q value < 0.05, Table S2). The top 20 significantly enriched pathways are shown in Figure 4B. The results revealed that DEGs were significantly enriched in two pathways related to cytochrome P450: “metabolism of xenobiotics by cytochrome P450” and “drug metabolism-cytochrome P450”. In addition, these DEGs were also significantly enriched in metabolic pathways, such as “glycine, serine, and threonine metabolism”, “amino sugar and nucleotide sugar metabolism”, “porphyrin and chlorophyll metabolism”, “pyruvate metabolism”, and “ascorbate and aldarate metabolism”. It is noteworthy that the “PPAR signaling pathway”, “porphyrin and chlorophyll metabolism”, “glycine, serine, and threonine metabolism”, and the cytochrome P450-related signaling pathways exhibit a close association with the redox state in vivo.

**Figure 5:**
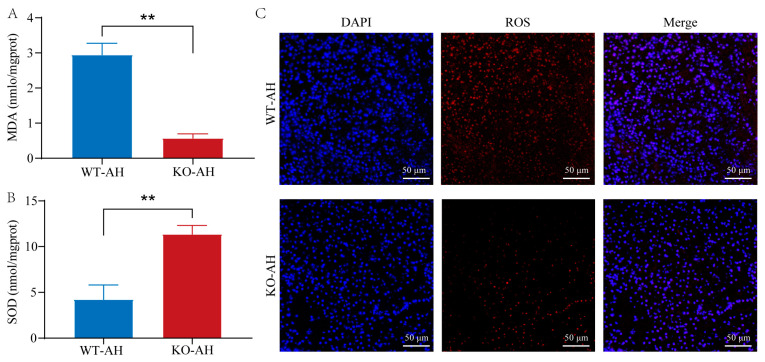
Effect of *il-6* mutation on oxidative stress triggered by *A. hydrophila* infection. (**A**) MDA and (**B**) SOD levels in liver tissues. Samples used for enzyme activity (biological replicates = 5, experimental repetitions = 3) were collected 12 h after *A. hydrophila* infection. Data were subjected to the Student’s *t*-test to identify the significance. ** *p* < 0.01. (**C**) Representative staining of ROS in liver sections. Samples were collected at 12 h after *A. hydrophila* infection. Frozen tissue sections were used for immunofluorescence detection of ROS. Red fluorescence corresponds to the presence of ROS, while blue fluorescence represents the cell nucleus. Scale bar = 50 μm. WT-AH: WT zebrafish treated with *A. hydrophila*; KO-AH: *il-6^−/−^* zebrafish treated with *A. hydrophila*.

To further verify whether *il-6* mutations affect redox levels during liver injury, we assessed oxidative stress status by measuring hepatic MDA levels (Figure 5A) and SOD activities (Figure 5B). The *il*-*6*^−/−^ zebrafish had lower MDA levels and higher antioxidant (SOD) activities compared with those of the WT zebrafish. This observation was also substantiated by ROS staining, which confirmed the reduced levels of hepatic oxidative stress in *il-6^−/−^* zebrafish (Figure 5C).

### 2.5. Expression and Purification of Recombinant Zebrafish IL-6 (rDrIL-6)

**Figure 6:**
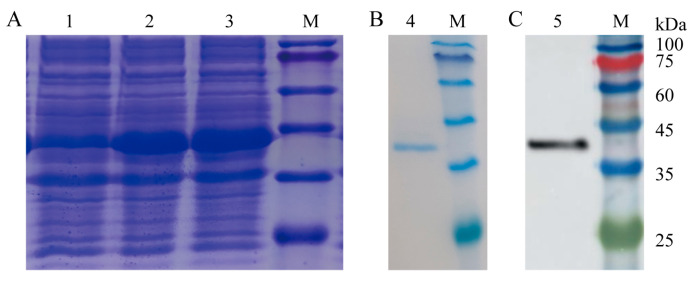
Expression and purification of recombinant zebrafish IL-6 (rDrIL-6) protein. (**A**) Analysis of rDrIL-6 protein by SDS-PAGE and WB. The utilized expression vector was pET-32a. The expression of the recombinant zebrafish IL-6 (rDrIL-6) protein was induced at 37 °C for 4 h with different concentrations of IPTG (1.0 and 1.5 mM). KDa: kilo Dalton; M: marker; 1: control group; 2: 1.0 mmol/L IPTG-induced group; 3: 1.5 mmol/L IPTG-induced group; (**B**) 4: assessment of purified rDrIL-6 protein by SDS-PAGE; (**C**) 5: confirmation of purified rDrIL-6 protein by WB. Anti-His antibody was used as a primary antibody.

The zebrafish *il*-*6* open reading frame (ORF) fragment was ligated into the pET-32a expression vector, and the recombinant His-tagged plasmid was transferred into the BL21(DE3) competent cells. Expressed rDrIL-6 was successfully confirmed by SDS-PAGE (Figure 6A). The SDS-PAGE (Figure 6B) and WB (Figure 6C) showed only one band between 35 kDa and 45 kDa after protein purification, which was consistent with the expected molecular weight of rDrIL-6. Meanwhile, rDrIL-6 possesses low hemolytic toxicity (Figure S2).

### 2.6. Effect of rDrIL-6 on A. hydrophila-Induced Liver Injury in il-6^−/−^ Zebrafish

**Figure 7:**
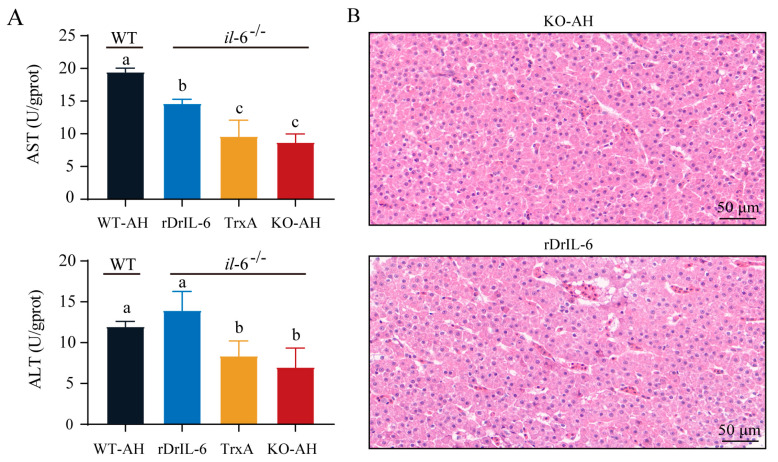
Effect of rDrIL-6 protein on liver injury induced by *A. hydrophila* infection in *il-6^−/−^* zebrafish. (**A**) The levels of ALT and AST in the liver. Samples used for enzyme activity (biological replicates = 5, experimental repetitions = 3) were collected 12 h after infection. The experimental data was subjected to the multifactor analysis of variance (ANOVA) to identify the significance. Values with the same letter superscripts represent no significant difference, while those with different letter superscripts represent significant differences (*p* < 0.05). (**B**) HE staining of liver tissues with different treatments. Samples were collected 12 h after *A. hydrophila* infection. Scale bar = 50 μm. WT-AH: WT zebrafish treated with *A. hydrophila*; rDrIL-6: *il-6^−/−^* zebrafish treated with a mixture of *A. hydrophila* and rDrIL-6 protein; TrxA: *il-6^−/−^* zebrafish treated with a mixture of *A. hydrophila* and control protein TrxA; KO-AH: *il-6^−/−^* zebrafish treated with *A. hydrophila*.

**Figure 8:**
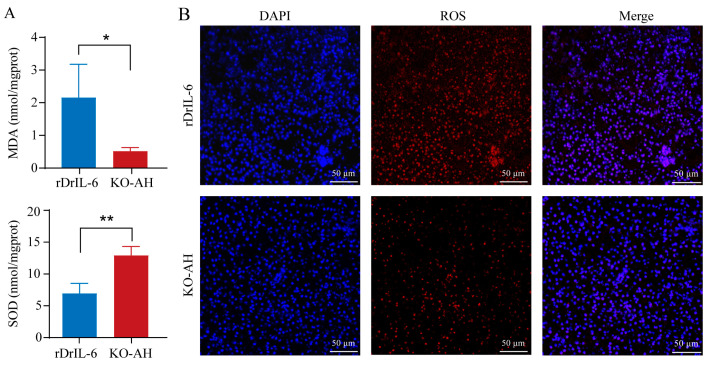
Effect of rDrIL-6 protein on liver oxidative stress in *il-6^−/−^* zebrafish post *A. hydrophila* infection. (**A**) MDA and SOD levels in liver tissues after *A. hydrophila* infection. Samples used for enzyme activity (biological replicates = 5, experimental repetitions = 3) were collected 12 h after infection. The experimental data were subjected to the Student’s t-test to identify the significance. Compared with the KO-AH group, the rDrIL-6 group showed significantly higher levels of MDA and lower SOD activities. * *p* < 0.05; ** *p* < 0.01. (**B**) Representative staining of ROS in liver sections. Frozen tissue sections were used for immunofluorescence detection of ROS. Red fluorescence corresponds to the presence of ROS, while blue fluorescence represents the cell nucleus. Scale bar = 50 μm. rDrIL-6: *il-6^−/−^* zebrafish treated with a mixture of *A. hydrophila* and rDrIL-6 protein; KO-AH: *il-6^−/−^* zebrafish treated with *A. hydrophila*.

In order to further substantiate the relationship between *il-6* deficiency and milder liver injury in *il-6^−/−^* zebrafish, rDrIL-6 and TrxA proteins were mixed with *A. hydrophila* and injected into *il-6^−/−^* zebrafish, respectively. Elevated ALT and AST levels suggested increased liver damage in the group treated with rDrIL-6 protein. At the same time, the control group treated with TrxA protein showed no discernible changes after *A. hydrophila* infection (Figure 7A). The results of HE staining showed that rdrIL-6 treatment aggravated liver injury after *A. hydrophila* infection in *il-6^−/−^* zebrafish (Figure 7B). Compared with the KO-AH group, the rDrIL-6 group showed significantly higher levels of MDA and lower SOD activities (Figure 8A). Similarly, the results of the ROS assay further confirmed that rDrIL-6 treatment increased the level of oxidative stress in *il-6^−/−^* zebrafish (Figure 8B).

## 3. Discussion

IL-6, as a pleiotropic immune cytokine, plays an important role in the immune response. In the mouse model of herpes simplex virus infection, IL-6 deficiency affected macrophage antiviral resistance, which could lead to increased morbidity and mortality [25]. *IL-6^−/−^* mice showed a significant reduction in neutrophil recruitment and lower mortality in the mouse model of acute pneumovirus infection [26]. These results suggest that IL-6 may play different roles in different infection models. In addition, fish, as the lowest vertebrate, exhibit an underdeveloped specific immune system compared to mammals. Therefore, fish rely more on non-specific immunity to counteract diverse pathogens [27], and IL-6, a typical cytokine, plays an important role in the non-specific immune response. In fish, recombinant IL-6 promotes splenocyte growth and induces the expression of pro-inflammatory factors such as TNF-α in monocyte macrophages [28,29]. In light of the scarcity of existing research concerning IL-6 in the context of piscine biology, in this study, *il-6^−/−^* zebrafish were constructed for the first time. This investigation primarily focused on elucidating the effects of *il-6* mutation on zebrafish response to *A. hydrophila* infection, with particular emphasis on the liver.

The elevated levels of AST and ALT are recognized as the typical markers of liver injury, implying an intensified hepatic injury state. In the present study, compared with WT zebrafish, AST and ALT levels were lower in the livers of *il-6*^−/−^zebrafish after infection with *A. hydrophila*, suggesting that *il-6* mutation in zebrafish attenuates *A. hydrophila*-induced liver injury. In the mouse model of concanavalin A-induced acute liver injury, reduction in IL-6 secretion has been shown to alleviate liver necrosis [30]. In alcoholic liver disease, inhibition of IL-6 has been demonstrated to reduce serum AST levels and attenuate ethanol-induced liver damage [11]. Similarly, in hepatic failure mice, suppression of the IL-6/STAT3 signaling pathway has been associated with a substantial alleviation in both liver injury and inflammatory responses, as well as a decrease in AST and ALT levels [31]. These studies suggest that reducing IL-6 attenuates liver injury induced by various factors.

To further investigate the mechanism of *il-6* mutation in attenuating *A. hydrophila*-induced liver injury, we performed RNA-seq of the liver tissues from WT and *il-6^−/−^* zebrafish after *A. hydrophila* infection. The results showed that the gene expression levels in the liver of WT and *il-6^−/−^* zebrafish were significantly different after *A. hydrophila* infection. GO enrichment analysis revealed that DEGs were significantly enriched in terms related to redox processes, such as copper ion transport, redox process, oxidoreductase activity, iron ion binding, and heme binding. Under normal physiological conditions, oxidation and reduction processes in organisms are maintained in a relatively steady state [32]. However, oxidative stress occurs when the antioxidant defense system is overwhelmed by oxidative processes, which is one of the ways in which the body eliminates invading pathogens. Nevertheless, excessive oxidative stress can lead to tissue damage. Previous studies have demonstrated that oxidoreductase activity, iron ion binding, and copper ion transport are closely related to oxidative stress [33,34]. Thus, *il-6* mutation may markedly affect the liver redox state during *A. hydrophila* infection.

Further, KEGG analysis showed that DEGs were significantly enriched in pivotal pathways, including the “PPAR signaling pathway”, “porphyrin and chlorophyll metabolism”, “glycine, serine and threonine metabolism”, and cytochrome P450-related signaling pathways. Notably, previous research has shown the complex association of these signaling pathways with oxidative stress. For example, echinacea purpurea could alleviate oxidative stress induced by hyperthyroidism through the PPAR signaling pathway [35]. Curcumin also attenuated oxidative stress induced by ROS in Nile tilapia by modulating the PPAR signaling pathway, thereby reducing hepatocyte damage [36]. Furthermore, porphyrin and chlorophyll metabolism were significantly enriched during oxidative defense in carp (*Cyprinus carpio* L.) after exposure to cadmium [35]. During oxidative stress triggered by chlorophyll deficiency in canola (*Brassica Napus*), ROS homeostasis was impaired and porphyrin and chlorophyll metabolism were also significantly enriched [37]. Abnormal activation of other oxidoreductases in the glycine, serine, and threonine metabolic pathways was observed in CoCl2-treated Caki-1 cells simulating hypoxia [38]. Similarly, in *Vibrio alginolyticus* and *Escherichia coli*, exogenous glycine disturbed redox homeostasis and increased oxidative stress by regulating this pathway [39]. Numerous studies have shown that oxidative stress is closely linked to excess ROS [40,41]. Increased ROS production induces mitochondrial dysfunction and plays a key role in oxidative stress [35]. This suggests that these pathways may be involved in ROS production or scavenging. Furthermore, *Flammulina velutipes* protected the liver by regulating the metabolism of xenobiotics through cytochrome P450 to alleviate the carbon tetrachloride-induced oxidative damage in mice [42]. Cytochrome P450 has been proven to be closely related to the formation of ROS in multiple species, such as mice [43], rats (*Rattus norvegicus*) [44], and hamsters (*Cricetinae*) [45]. Given these complex biochemical interactions, it is plausible that *il-6* mutation in zebrafish exerts a discernible influence on hepatic oxidative stress levels during *A. hydrophila* infection through the PPAR signaling pathway, cytochrome P450-related signaling pathway, and other intricate mechanisms that have not yet been elucidated.

Subsequently, we examined the effect of *il-6* mutation on liver redox levels in zebrafish infected with *A hydrophila*. MDA is the end product of lipid peroxidation and is considered to be an indicator of ROS-induced tissue damage [46]. SOD is a key antioxidant enzyme in the natural immune system to scavenge ROS [47]. Therefore, MDA and SOD are considered typical markers of oxidative stress. In this study, the effect of *il-6* mutation on *A. hydrophila*-induced oxidative stress levels in the zebrafish liver was assessed by measuring MDA levels and SOD activities. The results showed that compared with the WT zebrafish, MDA levels were significantly lower and SOD activities were significantly higher in the *il*-*6*^−/−^ zebrafish liver after *A hydrophila* infection. The results of ROS section staining again confirmed that *il-6* mutation reduced the ROS production induced by *A. hydrophila*. In oral squamous cell carcinoma cells, blocking NF-κB activation reduced ROS production, which was reversed by exogenous IL-6 treatment [48]. IL-6 can also stimulate ROS production in hepatoma cells [49]. *IL-6* knockdown significantly inhibited mitochondrial ROS production in skeletal muscle induced by cecal ligation and puncture, thereby attenuating skeletal muscle atrophy [12]. The present results are in accordance with these previous studies, indicating that the loss of *il-6* can inhibit the excessive accumulation of ROS to some extent. Excessive ROS can cause cell death and tissue damage [50,51]. Thus, the alleviation of oxidative stress induced by *A. hydrophila* may be one of the ways that *il-6* mutation attenuates liver injury. However, the mechanism of IL-6 regulation of oxidative stress remains to be further investigated.

## 4. Materials and Methods

### 4.1. Zebrafish Strain

The wild-type (WT) AB zebrafish strain used in this study was acquired from the Institute of Hydrobiology, Chinese Academy of Science (Wuhan, China). All experimental procedures involving zebrafish were approved by the animal care and use committee of Huazhong Agricultural University (approval number HZAUFI-2022-0023).

### 4.2. Generation of il-6^−/−^ Zebrafish by CRISPR/Cas9

The sgRNA target site of zebrafish *il-6* was designed by the CRISPR/Cas9 target online predictor (CCTop, http://crispr.cos.uni-heidelberg.de, accessed on 27 August 2021). The template of sgRNA was amplified by specific primers (Table S3), and the sgRNA was synthesized using a Transcript Aid T7 Hight Yield Transcription kit (Thermo Fisher Scientific, Waltham, MA, USA) according to the manufacturer’s instructions. The gene knockout experiment in zebrafish embryos was performed with reference to a previous report [52]. Genomic regions on either side of the sgRNA target site were amplified and sequenced to determine the genotype of the mutant. F1 was generated by backcrossing WT zebrafish with F0-positive zebrafish. Then, F2 was generated by self-breeding from F1 zebrafish with the same genotype (*il*-6^+/-^), and *il*-*6*^−/−^ zebrafish were obtained by screening.

### 4.3. A. hydrophila Infection and Samples Collection

*A. hydrophila* was kindly provided by Associate Professor Yi Luo from the College of Fisheries, Huazhong Agricultural University [53]. Only adult zebrafish (4 months of age, 0.35 ± 0.05 g) were used in this study. To determine the optimal injection concentration of *A. hydrophila*, different concentrations of 1 μL *A. hydrophila* (1 × 10^4^, 1 × 10^5^, 1 × 10^6^ CFU/mL) were separately injected intraperitoneally into WT zebrafish (n = 20), and survival curves were plotted (Figure S3). At the same time, 1 μL PBS was injected intraperitoneally in the negative control group. The half-lethal concentration was determined to be 2.5 × 10^5^ CFU/mL and used for subsequent experiments.

We injected equal amounts of *A. hydrophila* (1 μL) in WT (n = 120) and *il-6^−/−^* zebrafish (n = 120), named WT-AH and KO-AH, respectively. Samples used for transcriptome sequencing (biological replicates (n = 5), experimental repetitions (N = 3)), and enzyme activity (n = 5, N = 3) were collected at 0 h and 12 h after *A. hydrophila* infection. The collected livers were stored at −80 °C. In addition, zebrafish livers were randomly collected for HE staining in each group.

To further test the effect of recombinant IL-6 protein on *il-6^−/−^* zebrafish infected with *A. hydrophila*, 180 *il-6^−/−^* zebrafish were randomly and equally divided into 3 groups (n = 60). One *il-6^−/−^* zebrafish group was injected intraperitoneally with a mixture of rDrIL-6 (1 μL per zebrafish, 10 ng/μL) and *A. hydrophila* (1 μL) and named the rDrIL-6 group. At the same time, rdrIL-6 was replaced with equal amounts of TrxA (labeled protein [54]) and PBS, which were named the TrxA group and KO-AH group, respectively. In addition, a group of WT zebrafish (n = 60) intraperitoneally injected with a mixture of PBS and *A. hydrophila* was named the WT-AH group. After 12 h of treatment, we collected livers for enzyme activity assays (n = 5, N = 3), HE staining, and ROS staining.

### 4.4. RNA Extraction, Library Construction, and RNA-Sequencing

Six RNA-seq liver libraries were constructed for transcriptome analysis. Total RNA from liver tissues was extracted using RNAiso Plus (Takara, Japan), following the manufacturer’s instructions. The integrity and concentration of RNA was measured by an Agilent 2100 Bioanalyzer (Agilent Technologies, Inc., Santa Clara, CA, USA). An NEBNext Poly (A) mRNA Magnetic Isolation Module (NEB, E7490) was used to isolate mRNA. The cDNA library was constructed using the NEBNext Ultra RNA Library Prep Kit for Illumina (NEB, E7530) and NEBNext Multiplex Oligos for Illumina (NEB, E7500) according to the manufacturer’s instructions. Finally, the constructed cDNA library was sequenced by the Illumina NovaSeq6000 sequencing platform.

### 4.5. RNA-Seq Data Analysis

The clean data were obtained by removing adapter sequences and low-quality reads from raw data through an in-house perl script. The Q20, Q30, GC-content, and sequence duplication levels of the clean data were also calculated. The clean reads were mapped to the zebrafish reference genome (GeneBank: GCA_000002035.4) using Hisat2.0.4 software [55]. Then, the aligned clean data were assembled by StringTie [56]. Fragments Per Kilobase of transcript per Million fragments mapped (FPKM) were calculated based on the read count and length of the gene [57].

DESeq2 was used to analyze the DEGs [58]. Genes with an adjusted fold change ≥ 2 and false discovery rate (FDR) < 0.01 found by DESeq2 were assigned as differentially expressed. The clusterProfiler packages based on Wallenius non-central hyper-geometric distribution were used for GO enrichment analysis of DEGs. The KOBAS database and clusterProfiler4.4.4 software were used to test the statistical enrichment of DEGs in KEGG pathways.

### 4.6. Fluorescent Quantitative Real-Time PCR (qPCR)

qPCR was used to validate the results of high-throughput RNA-seq. The cDNA was synthesized using the HiScript^®^ II Q RT SuperMix for qPCR (+gDNA wiper) (R223-01, Vazyme Biotech Co., Ltd, Nanjing, China) according to the manufacturer’s instructions. Primers were designed using Primer Premier 5 (PREMIER Biosoft International, Palo Alto, CA, USA), and the gene-specific primers are listed in Table S3. To test primer specificity, Taq DNA polymerase (CWBIO) was used for amplification and the products were analyzed by 2% agarose gel electrophoresis. Before qPCR assays, all cDNA concentrations were adjusted to 50 ng/mL and stored at −80 °C. qPCR was performed using the LightCycler^®^ 480 SYBR Green I Master (Roche, Basel, Switzerland) on a Roche LightCycler^®^ 480 System (Roche, Basel, Switzerland) following the manufacturer’s instructions. The qPCR mixture contained 1 μL of cDNA sample, 7.4 μL of nuclease-free water, 10 μL of LightCycler^®^ 480 SYBR Green I Master Mix, and 0.8 μL of each upstream and downstream PCR primer. qPCR was conducted using the following program: 95 °C for 5 min, 40 cycles of 95 °C for 5 s, 60 °C for 20 s, and 72 °C for 20 s, followed by melting curve determination from 65 °C to 95 °C to verify the amplification of a single product. The internal control was *18s rRNA*, and the relative mRNA expression level was calculated with the 2^−ΔΔCT^ method. qPCR data were presented as mean ± standard error of the mean (SEM).

### 4.7. Protein Expression and Purification

The ORF sequence of the *il-6* gene was amplified using gene-specific primers (Table S3) for prokaryotic expression. The resulting amplicon was digested with *Xho* I and *EcoR* I and then inserted into the multiple cloning sites (MCS) of the pET-32a vector. The recombinant plasmid pET-32a-IL-6 was transformed into Trelief™ 5α recombinant competent cells (Tsingke Biological Technology, Beijing, China) and then transformed into *E. coli* BL21 (DE3) competent cells (Tsingke Biological Technology, Beijing, China). The expression of the recombinant zebrafish IL-6 (rDrIL-6) protein was induced at 37 °C for 4 h with different concentrations of IPTG (1.0 and 1.5 mM). Subsequently, the rDrIL-6 protein was purified by Ni-NTA (nitrilotriacetic acid) affinity chromatography (Sangon, Shanghai, China) according to the manufacturer’s instructions. The purified intact protein was confirmed via sodium dodecyl sulfate polyacrylamide gel electrophoresis (SDS-PAGE) and Western blot (WB) with anti-His antibody (1:2000, ABclonal), and an endotoxin detection assay was confirmed without LPS contamination in this recombinant protein (<0.2 endotoxin units). The purified protein was quantitated using the Bradford protein quantitation assay by Nanodrop 2000 (Thermo Fisher Scientific, Waltham, MA, USA).

### 4.8. HE Staining

HE staining was performed as previously described [59]. In short, livers were removed and immediately placed in fixative (10% formalin), dehydrated, embedded, and cut into 4 μm sections. Subsequently, the samples were mounted on slides coated with aminopropyl triethoxysilane. Paraffin was removed by xylene and subjected to HE staining. Images were taken using CaseViewer2.4 software.

### 4.9. ROS Staining

Livers were collected for ROS staining [60]. Briefly, the livers were dehydrated through different concentrations of sucrose solution after fixation. They were then subjected to OTC embedding and sectioning. Sections were incubated with ROS (D7008, Sigma-Aldrich, Shanghai, China) and DAPI (G1012, Servicebio, Wuhan, China) to avoid exposure to light and then washed with PBS, respectively. Images were acquired through a fluorescence microscope (Nikon Eclipse C1, Tokyo, Japan).

### 4.10. Measurement of Liver Biochemical Indicators

The liver tissues from zebrafish were collected and rapidly frozen at -80 °C for further analysis. Subsequently, 9 volumes of homogenization medium (phosphate buffer solution, pH = 7.4) were added. The mixture was then subjected to centrifugation in a refrigerated centrifuge (4 °C, 12,000 rpm, 15 min). The supernatant was then collected for subsequent determination of the enzyme activity index. Biochemical indicators, including alanine aminotransferase (ALT), aspartate aminotransferase (AST), superoxide dismutase (SOD), and malondialdehyde (MDA) were examined using commercial kits (Nanjing Jiancheng Bioengineering Institute, Nanjing, China).

### 4.11. Statistical Analysis

Data are presented as mean ± SEM of three repeated experiments and all statistical analyses were performed using the SPSS 26.0 package (IBM, New York, NY, USA). The experimental data of two groups were subjected to the Student’s *t*-test to identify the significance. *p* < 0.05 indicated significant difference (*), and *p* < 0.01 indicated extremely significant difference (**). The experimental data of four groups were subjected to the multifactor analysis of variance (ANOVA) to identify the significance. Values with the same letter superscripts represent no significant difference, while those with different letter superscripts represent significant differences (*p* < 0.05).

## 5. Conclusions

In this study, an *il-6* knockout zebrafish line was obtained. GO and KEGG analyses showed that DEGs in the liver of *il-6^−/−^* zebrafish compared with WT zebrafish were mainly enriched in redox terms and pathways. MDA levels, SOD activities, and ROS staining showed that *il-6* mutation attenuated the oxidative stress induced by *A. hydrophila* infection in the liver. In addition, *il-6^−/−^* zebrafish exhibited milder liver injury after *A. hydrophila* infection compared with WT zebrafish. In summary, this study provides evidence that *il-6* mutation attenuates oxidative stress during inflammation, thereby reducing liver injury, and offers information for the prevention and treatment of fish bacterial diseases in the future.

## Data Availability

All datasets generated for this study are included in the article and the Supplementary Materials.

## Supplementary Materials

The supporting information can be downloaded at https://www.mdpi.com/article/10.3390/ijms242417215/s1.
