# Supplementary material for: IL-6 Mutation Attenuates Liver Injury Caused by Aeromonas hydrophila Infection by Reducing Oxidative Stress in Zebrafish
Source: Int J Mol Sci. 2023 Dec 7;24(24):17215. doi: 10.3390/ijms242417215 (PMC10743878; doi:10.3390/ijms242417215)
Supplement: Supplementary file 1 [file ijms-24-17215-s001.zip › ijms-2712484-supplementary.pdf]

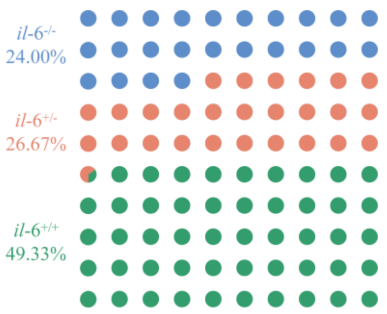

Figure. S1 Statistics of the number of different genotypes zebrafish. The count of adult fish belonging to the categories of homozygous (*il-6*$^{-/-}$), heterozygous (*il-6*$^{+/-}$), and wild type (*il-6*$^{+/+}$) in F2.

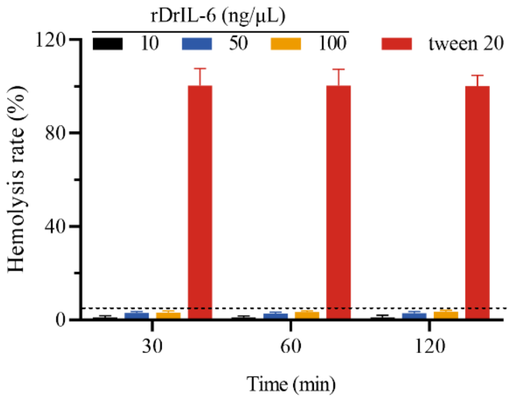

Figure. S2 Hemolysis study of rDrIL-6. Hemolytic activity of each group was detected with 2% erythrocytes for 2 h at 37 ◦C. Tween 20 was used as a positive control.

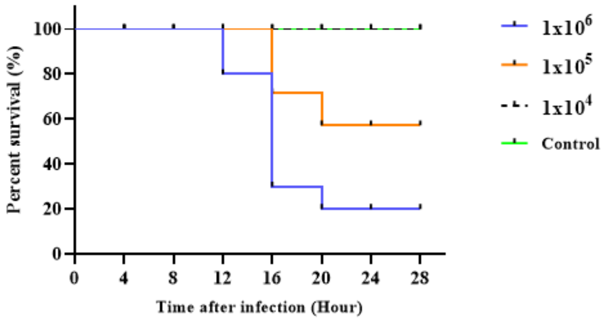

Figure. S3 Survival curves. Different concentrations of *A. hydrophila* were injected into WT zebrafish. PBS was used as negative control group.

Table S1 Sequencing data statistics

Samples	Clean reads	Clean bases	Q20 (%)	Q30 (%)	GC (%)
WT-AH-1	24087734	7197071884	97.76	93.50	46.48
WT-AH-2	22431348	6709101456	98.42	94.98	46.78
WT-AH-3	26984273	8065064692	97.49	92.85	47.16
KO-AH-1	23529819	7039470312	97.47	92.76	46.98
KO-AH-2	21851054	6498401222	97.79	93.71	45.15
KO-AH -3	23785409	7113612090	97.33	92.47	48.01

WT-AH: WT zebrafish were treated with *A. hydrophila*; KO-AH: *il-6*$^{-/-}$ zebrafish were treated with *A. hydrophila*.

Table S2 Significantly enriched KEGG pathway

Pathway	Gene Ratio	q value	Gene number
Ribosome	3.85%	5.66E-14	66
Steroid biosynthesis	1.11%	3.99E-09	19
Carbon metabolism	3.73%	2.51E-08	64
PPAR signaling pathway	2.57%	2.71E-07	44
Pentose and glucuronate interconversions	1.57%	7.79E-05	27
Porphyrin and chlorophyll metabolism	1.63%	7.79E-05	28
Terpenoid backbone biosynthesis	0.93%	7.79E-05	16
Steroid hormone biosynthesis	1.87%	9.25E-05	32
Glyoxylate and dicarboxylate metabolism	1.22%	9.25E-05	21
One carbon pool by folate	0.76%	0.0002298	13
Amino sugar and nucleotide sugar metabolism	1.75%	0.0002413	30
Glycine, serine and threonine metabolism	1.22%	0.0002413	21
Proteasome	1.34%	0.0002924	23
Ascorbate and aldarate metabolism	1.28%	0.0003162	22
Metabolism of xenobiotics by cytochrome P450	1.69%	0.0003419	29
Biosynthesis of amino acids	2.10%	0.0003419	36
Pyruvate metabolism	1.40%	0.0006344	24
Protein processing in endoplasmic reticulum	4.02%	0.0006930	69
Drug metabolism - cytochrome P450	1.63%	0.0007329	28
Drug metabolism - other enzymes	2.04%	0.0019157	35
Arginine and proline metabolism	1.57%	0.0020473	27
Fatty acid biosynthesis	0.76%	0.0067974	13
p53 signaling pathway	2.04%	0.0072303	35
Prion disease	0.41%	0.0078607	7
Retinol metabolism	1.69%	0.0083972	29
Pentose phosphate pathway	0.99%	0.0136290	17
Glycosylphosphatidylinositol (GPI)-anchor	0.58%	0.0150207	10

biosynthesis			
Complement and coagulation cascades	0.47%	0.0174599	8
Pantothenate and CoA biosynthesis	0.58%	0.0188271	10
Cysteine and methionine metabolism	1.22%	0.0190987	21
Fatty acid metabolism	1.52%	0.0190987	26
Systemic lupus erythematosus	0.41%	0.0232069	7
Starch and sucrose metabolism	0.93%	0.0232331	16
ABC transporters	1.17%	0.0245442	20
Propanoate metabolism	0.87%	0.0272690	15
Phagosome	4.02%	0.0287981	69
Fatty acid degradation	1.05%	0.0301899	18
Vitamin digestion and absorption	0.23%	0.0352759	4
Cholesterol metabolism	0.23%	0.0352759	4
Protein export	0.52%	0.0365142	9
Synthesis and degradation of ketone bodies	0.41%	0.0368678	7
Hepatitis C	0.47%	0.0416431	8
Adipocytokine signaling pathway	1.69%	0.0440273	29

Analysis of *il-6*$^{-/-}$ and WT zebrafish liver transcriptome KEGG enrichment following *A. hydrophila* infection. q value ≤ 0.05 indicated significantly enriched KEGG pathway.

Table S3 Primers used in this study

**Primer name**	**Sequence (5′-3′)**	**Application**
IL-6-sg-F	GTAATACGACTCACTATAGGCAGCGGTCTGAAGGTTTG GTTTTAGAGCTAGAAATAGC	Amplified sgRNA
IL-6-sg-R	AAAAGCACCGACTCGGTGCC	template
apoeb-F	CAGATGACCCCATACGCCTC	qPCR
apoeb-R	GGCGTTTCTTCAGTTTGCGT	
dazap2-F	TCTAGTGTCCCGATGGCGTA	qPCR
dazap2-R	CAGATGGTGTAACCACCGCT	
aldh1l1-F	GGTCCCCAAAACCACAAAGC	qPCR
aldh1l1-R	GCCCAAAAGACTCCTCGACA	
abcc2-F	GGTTACCTGTATGCGGTGCT	qPCR
abcc2-R	CCGGGAGTCATTGGACACAA	
ctnnb2-F	GCTCCACTCACTGAACTGCT	qPCR
ctnnb2-R	CGTCTGCTCTGTATGCGAGT	
aclya-F	GCGGGAACACCCTACAAGAA	qPCR
aclya-R	ATCCTGGACCAGTCTCTCCC	
cyp51-F	GATGGCCAAAACCCCTCAGA	qPCR
cyp51-R	CCGAATGGGATGTAGGCGAA	

dpm3-F	GCTGGCATTGAATAGTAGGTGG	qPCR
dpm3-R	TCGGCCATGCAAGCTCTTTA	
lrpap1-F	CTCACTGATTGGCTGGGGTT	qPCR
lrpap1-R	CCCACATGTTTGTGGGAAGC	
tmco1-F	AATGCTGACGCTGCTACAGT	qPCR
tmco1-R	CGGTACACCAGAACCCATGT	
18s-F	CACTGCACAGAGGTCTCGTT	qPCR
18s-R	TTGGTGTCAGCCCTTACGTC	
IL-6-32a-F	CCGGAATTCATGCCATCCGCTCAGAAAACA	plasmid
IL-6-32a-R	CCGCTCGAGTTAGTTCTTGTCAGCGCTGCCC	construction
